# Incremental Predictive Value of Post-Baseline Surgery and the Impact of Temporal Coding on Mortality Associations in Infective Endocarditis: A Temporally Validated Cohort Study

**DOI:** 10.3390/diagnostics16193209

**Published:** 2026-10-02

**Authors:** Banu Karaca, Aykan Çelik, Mert İnan, Berke Ege, Mustafa Karaca

**Affiliations:** 1 Department of Infectious Disease, Ataturk Training and Research Hospital, Faculty of Medicine, Izmir Katip Celebi University, Izmir 35360, Türkiye; 2 Department of Cardiology, Ataturk Training and Research Hospital, Faculty of Medicine, Izmir Katip Celebi University, Izmir 35360, Türkiye

**Keywords:** infective endocarditis, mortality prediction, time-dependent covariate, cardiac surgery, immortal-time bias, temporal validation, prognostic modeling

## Abstract

**Background:** Risk prediction in infective endocarditis (IE) may be compromised when treatments occurring after the initial evaluation are incorporated as though they were baseline predictors. Surgical treatment is inherently time-dependent, yet subsequent surgery has been incorporated as a static predictor in contemporary prognostic models, potentially affecting both predictive performance and treatment–outcome associations. We evaluated whether adding subsequent surgery improves mortality prediction beyond information obtained during the initial index-episode evaluation and examined how temporal representation of surgery influences its association with mortality. **Methods:** We retrospectively studied 293 consecutive patients with IE. The primary outcome was 180-day all-cause mortality. Model A incorporated seven prespecified predictors obtained during the initial index-episode evaluation: age, NYHA functional class, hemoglobin, blood urea nitrogen, left ventricular ejection fraction, severe regurgitation, and C-reactive protein. Model B additionally incorporated a binary indicator of surgery within 180 days. Predictive performance was assessed using time-dependent AUC, Brier score, 1000-replicate bootstrap cross-validation, and temporal validation using 2008–2017 as the derivation period and 2018–2026 as the validation period. Surgery–mortality associations were compared using fixed baseline and time-dependent exposure definitions. **Results:** Ninety-two patients (31.4%) died within 180 days, and 122 (41.6%) underwent surgery within 180 days. In the complete-case cohort (*n* = 278), Model A had an apparent AUC of 0.792 and a bootstrap cross-validated AUC of 0.759. Adding future surgery produced no detectable improvement in apparent discrimination (AUC 0.793 vs. 0.792; ΔAUC +0.001, 95% CI −0.018 to +0.019) or after bootstrap cross-validation (AUC 0.749 vs. 0.759; ΔAUC −0.010, 95% CI −0.057 to +0.014). In temporal validation, AUCs were 0.731 for Model A and 0.733 for Model B. Mean predicted 180-day mortality was 27.3% versus an observed Kaplan–Meier risk of 34.7% (95% CI 25.5–42.8%); the observed-minus-predicted difference was +7.5 percentage points (bootstrap 95% CI −2.4 to +18.8). Fixed baseline coding yielded an adjusted HR of 0.71 (95% CI 0.44–1.17) in all 278 complete cases and 0.58 (95% CI 0.34–0.99) after excluding five same-day surgery/death records; in the corresponding 273-patient matched sample, time-dependent coding yielded an HR of 1.44 (95% CI 0.83–2.49). Exploratory phase-specific analyses suggested higher mortality during the early postoperative period, but the magnitude was sensitive to same-day event handling. **Conclusions:** In this cohort, adding a binary indicator of subsequent surgery to the prespecified seven-predictor baseline model produced no detectable improvement in 180-day mortality prediction. In contrast, the estimated surgery–mortality association changed substantially according to temporal exposure coding. Prediction models should respect the intended prediction time, while observational analyses of post-baseline treatments should explicitly account for treatment timing.

## 1. Introduction

Infective endocarditis (IE) remains a life-threatening disease despite advances in antimicrobial therapy, cardiac imaging, multidisciplinary care, and surgical management. Contemporary guidelines emphasize early identification of patients at high risk of complications and recognize heart failure, uncontrolled infection, and prevention of embolic events as the principal indications for surgery [1]. Because both disease severity and treatment pathways vary substantially between patients, reliable risk stratification at the time of diagnosis remains clinically important.

A variety of clinical, biomarker-based, and surgical risk models have been proposed for infective endocarditis. A systematic review published in 2023 identified substantial heterogeneity among available risk scores and highlighted limitations related to model development, external validation, and transportability [2]. More recent investigations have incorporated inflammatory biomarkers and machine-learning approaches, while a recently developed European model has undergone external validation for early mortality prediction in left-sided infective endocarditis [3,4,5]. Nevertheless, differences in study populations, prediction horizons, and predictor availability complicate comparisons between existing models and emphasize the importance of defining the intended prediction time.

An additional methodological challenge arises when a treatment delivered after diagnosis is incorporated into a model intended to estimate prognosis from the time of diagnosis. This issue is not limited to a single modeling approach. A 2022 nomogram developed for early identification of in-hospital mortality included absence of surgical treatment as an independent predictor and allowed estimated mortality to vary according to the surgical treatment option [6]. More recently, an externally validated 2025 nomogram for in-hospital mortality retained surgery among five final predictors, while a 2026 model for 1-year mortality similarly incorporated surgical treatment as a prognostic factor [7,8]. In these settings, eventual receipt of surgery may not be known at the intended prediction time and may therefore introduce information that reflects not only underlying prognosis but also subsequent treatment selection and survival until treatment.

This concern was particularly explicit in a recent machine-learning study of 444 patients with IE that developed models for 6-month mortality using nine predictors, including receipt of surgical intervention; surgery emerged as the most influential feature in the final model and was associated with lower predicted mortality. Importantly, surgery was modeled as a baseline covariate despite occurring after diagnosis [5]. The authors subsequently acknowledged that this representation did not account for the time-dependent nature of surgery and could introduce immortal-time bias, and they specifically identified time-dependent covariate modeling as an important direction for future investigation [5]. Their sensitivity analysis also showed that removal of surgery did not significantly impair overall model performance [5].

The importance of temporal treatment allocation in infective endocarditis has been recognized previously. A recent methodological discussion specifically highlighted immortal-time bias in observational comparisons of cardiac surgical treatment, while contemporary nationwide data have further demonstrated the complexity of surgical selection and mortality associations among patients with infective endocarditis [9,10]. These observations emphasize the need to distinguish treatment timing from baseline prognostic information. However, analyses addressing the association between surgery and mortality do not directly establish whether including future surgery improves the predictive performance of a model intended for use during the initial clinical evaluation.

These observations raise two related but distinct questions. First, does incorporating knowledge of subsequent surgery improve mortality prediction beyond information obtained during the initial index-episode evaluation? Second, does treating eventual surgery as though it were a baseline characteristic materially alter the estimated association between surgery and mortality? The methodological contribution of the present study was therefore not to demonstrate immortal-time bias per se, which has previously been recognized in IE, but to examine these two issues within the same cohort and analytic framework. Because our objective was methodological rather than to derive or validate a new clinical risk score, we used a deliberately parsimonious baseline model composed of routinely available variables obtained during the initial index episode as a common reference for assessing the incremental contribution of subsequent surgery. We evaluated 180-day mortality using this baseline model, quantified the incremental predictive contribution of subsequent surgery, and assessed model performance using bootstrap internal validation and temporal validation. In parallel, using the same baseline adjustment set, we compared fixed baseline and time-dependent representations of surgery and explored temporal variation in the surgery–mortality association.

## 2. Methods

### 2.1. Study Design and Population

This retrospective, single-center cohort study included consecutive adult patients diagnosed with infective endocarditis at a tertiary center between 2008 and 2026. Infective endocarditis was diagnosed according to the modified Duke criteria, based on the available clinical, microbiological, and echocardiographic findings [11]. Definite-versus-possible modified Duke classification was not retained as a separate structured variable in the retrospective dataset, and these categories were not retrospectively reassigned from incomplete structured components; therefore, reliable category-specific counts could not be reported. Patients were identified from institutional clinical records. The index date (time zero) was defined as the beginning of the index hospital episode at which IE was first clinically recognized. When microbiological, echocardiographic, or operative findings subsequently strengthened or confirmed the diagnosis during the same episode, follow-up remained anchored to this initial clinical recognition date rather than to the later confirmation date. No patient underwent cardiac surgery before or on the index date; the earliest recorded cardiac surgery occurred one day after time zero. Patients were followed from the index date until death, last documented follow-up, or the end of the prespecified prediction horizon, as appropriate.

**Figure 1:**
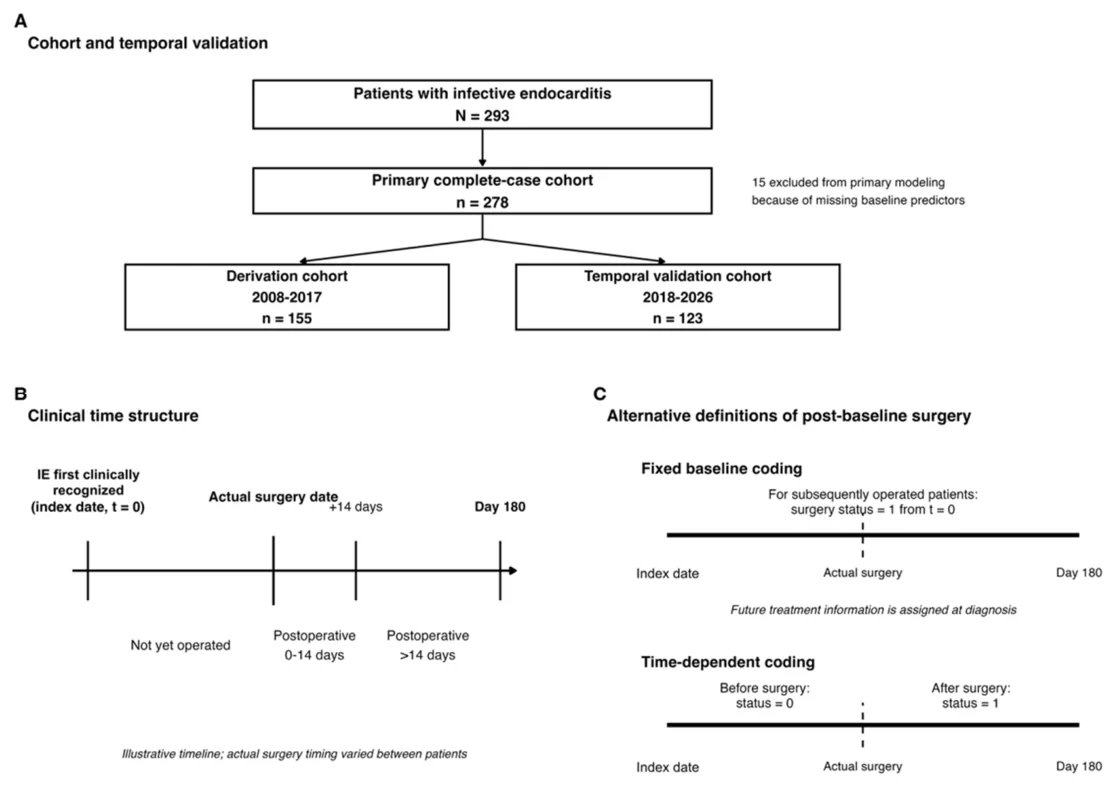
Study design and temporal representation of surgical treatment. (**A**) Cohort structure for the primary analysis and temporal validation. Of 293 patients with infective endocarditis, 278 had complete data for the prespecified baseline predictors. The complete-case cohort was divided chronologically into a derivation cohort from 2008–2017 (*n* = 155) and a temporal validation cohort from 2018–2026 (*n* = 123). (**B**) Illustrative clinical timeline showing initial clinical recognition of infective endocarditis as time zero, subsequent surgery occurring at a patient-specific post-baseline time, and postoperative states divided into the first 14 days and the period thereafter. (**C**) Contrast between fixed baseline coding, in which eventual surgery is retrospectively assigned from the index date onward for patients who subsequently undergo surgery, and time-dependent coding, in which exposure status changes only at the actual procedure date.

The full study cohort comprised 293 patients. The primary prediction analyses were performed using patients with complete data for all prespecified baseline predictors. Patients were not excluded on the basis of subsequent treatment, including surgery, because surgical management was considered a post-baseline clinical event rather than an eligibility criterion. For temporal validation, the complete-case cohort was divided chronologically into an earlier derivation period (2008–2017) and a later validation period (2018–2026) (Figure 1). No random train–test split was used.

The present cohort partially overlaps with a previously published cohort from the same institution, but it was reconstructed through a new review of institutional records rather than by simply extending the earlier final analytic dataset [4]. The previous study examined admission inflammatory biomarkers and long-term mortality among patients hospitalized between 2008 and 2021 and excluded patients who died during the index hospitalization. For the present study, eligibility, index dates, treatment information, and follow-up were re-reviewed, and early or in-hospital death was not an exclusion criterion because 180-day all-cause mortality from the index IE episode was the primary outcome. Definitive patient-level membership of the previous final 204-patient analytic cohort was not retained in the archived source records; therefore, the exact number of patients shared by the two final analytic cohorts, and the exact number of the previously excluded in-hospital deaths represented in the present cohort, could not be reconstructed reliably.

### 2.2. Outcome Definition and Follow-Up

The primary outcome was 180-day all-cause mortality. Secondary prediction horizons were 30-day and 90-day all-cause mortality. Vital status and dates of death were verified through the national health database when reliable national registry linkage was available. For foreign nationals for whom national database linkage was unavailable or unreliable, vital status was determined from institutional and follow-up records. Patients with shorter documented follow-up and no reliably ascertainable vital status beyond their last clinical contact were censored at their last known follow-up. The final documented follow-up date was 28 July 2026. Surgical treatment information was obtained from institutional records and follow-up documentation and included procedures performed at other centers when these were documented during follow-up. Among the 278 complete-case patients, 17 foreign-national patients were censored alive at their last documented clinical follow-up before 180 days because reliable linkage to the national vital-status database was unavailable (8 in the 2008–2017 derivation period and 9 in the 2018–2026 validation period). Of these 17 patients, 8 had undergone surgery before censoring and were classified as surgically treated. The remaining 9 had no surgery documented before their last follow-up and were therefore classified as having no observed surgery; however, surgery occurring after censoring but before day 180 could not be excluded. To address this uncertainty, the fixed-horizon sensitivity analysis excluded patients without ascertainable 180-day status.

For horizon-specific prediction analyses, surgery was defined according to the same prediction horizon: surgery occurring within 30 days for 30-day mortality, within 90 days for 90-day mortality, and within 180 days for the primary 180-day analysis. This horizon-matched definition was used to avoid assigning treatment information occurring beyond the outcome-prediction window.

### 2.3. Candidate Predictors and Baseline Reference Model

The primary baseline prediction model was prespecified as a parsimonious reference model using variables obtained during the initial index-episode evaluation. Predictor selection was based on clinical relevance, routine availability in the study cohort, representation of complementary prognostic domains, and the need to maintain an appropriate model dimension relative to the number of observed events. The seven predictors were age, NYHA functional class, hemoglobin, blood urea nitrogen (BUN), left ventricular ejection fraction (LVEF), severe valvular regurgitation, and C-reactive protein (CRP), representing demographic risk, functional status, hematologic and inflammatory burden, renal dysfunction, and cardiac structural or functional impairment. For Model A, predictor values were restricted to the initial assessment of the index IE episode. Age was defined at the index date, and NYHA functional class reflected the initial clinical evaluation. Hemoglobin, BUN, and CRP were taken from the first laboratory assessment at the beginning of the index hospitalization. LVEF and severe valvular regurgitation were derived from the first echocardiographic examination performed as part of the initial admission assessment on the index date (time zero). No follow-up or postoperative echocardiographic measurements were incorporated into Model A. For reproducibility, the complete Model A regression coefficients, predictor coding, and estimated uncentered baseline survival at 180 days are provided in Supplementary Table S1. The objective was not to derive or propose a comprehensive new IE risk score, nor to identify an optimal set of mortality predictors. Rather, Model A was designed as a clinically interpretable baseline reference model against which the incremental predictive contribution of subsequent surgery could be evaluated while keeping the remaining predictor set identical. Established IE and operative risk scores were therefore not used as the primary reference model, because the purpose of the analysis was not to compare competing clinical scores but to isolate the incremental contribution of a post-baseline treatment variable within a common baseline prediction framework. This approach was consistent with contemporary prediction-model guidance emphasizing explicit definition of the intended prediction time, clinically justified predictor specification, avoidance of data-driven variable selection when the objective is not model derivation, and transparent reporting of model parameters [12,13].

For primary modeling, age was expressed per 10-year increase, BUN per 10-mg/dL increase, and LVEF per 5-percentage-point increase. NYHA functional class was modeled as an ordinal variable, and CRP was transformed as log(1 + CRP) because of its markedly right-skewed distribution. Severe regurgitation was modeled as a binary indicator. All prespecified predictors were retained in the multivariable model irrespective of their individual statistical significance; no univariable screening or automated variable-selection procedure was performed.

This baseline-only model was designated Model A and was intended to represent the initial index-episode evaluation. A second model (Model B) contained the identical baseline predictor set plus subsequent surgery within the relevant prediction horizon. Model B was not intended as a deployable prediction model for use during the initial index-episode evaluation; rather, it was used as a methodological comparator to quantify whether incorporating post-baseline treatment information altered predictive performance.

### 2.4. Temporal Representation of Surgery

For the primary exposure definition, cardiac surgery included procedures recorded as surgical interventions for IE, including surgically performed CIED system extraction. CIED system extraction performed without a recorded cardiac surgical procedure was not classified as cardiac surgery. Surgical treatment was represented in two distinct ways depending on the analytic question (Figure 1). First, for the fixed-exposure comparison, eventual surgery within 180 days was coded as a conventional binary covariate present from time zero. This representation intentionally mirrors the common but temporally problematic practice of treating a post-baseline intervention as though it were known at baseline [14].

Second, surgery was modeled as a time-dependent exposure using the recorded procedure date. Each patient contributed person-time to the not-yet-operated state until the date of surgery and, if surgery occurred, to the postoperative state thereafter. Thus, patients were not classified as surgically exposed during the preoperative interval. This approach prevents preoperative survival time from being retrospectively attributed to the surgical state [14].

Five patients had surgery and death recorded on the same calendar day, for which within-day ordering was unavailable. These patients were excluded from the primary same-sample fixed-versus-time-dependent comparison, and alternative same-day conventions were examined in sensitivity analyses as described below.

### 2.5. Exploratory Postoperative Phase-Specific Analysis

To examine whether the association between surgical status and mortality varied over postoperative time, the time-dependent surgical state was further divided into three mutually exclusive states: not yet operated, postoperative days 0–14, and more than 14 days after surgery. The not-yet-operated state served as the reference category. The 14-day division was used to distinguish the immediate postoperative period from later postoperative follow-up and was retained consistently in this exploratory phase-specific analysis.

Adjusted phase-specific Cox models included the same baseline covariates as the primary prediction model. A formal contrast was used to compare the hazard associated with the postoperative 0–14-day state directly with that of the >14-day postoperative state. Because surgical allocation was not randomized and treatment decisions were influenced by clinical status, all surgery-related hazard ratios were interpreted as observational associations rather than causal treatment effects.

### 2.6. Primary Survival Modeling

Associations with 180-day mortality were estimated using Cox proportional-hazards regression. Model A was the baseline Cox model and included the seven prespecified predictors obtained during the initial index-episode evaluation. Model B contained the same predictor set and additionally incorporated surgery within 180 days as a fixed binary covariate. For surgery-association analyses, fixed-baseline and time-dependent exposure specifications were compared using identical patient samples and identical baseline adjustment variables whenever possible. Hazard ratios (HRs) are reported with 95% confidence intervals (CIs).

The proportional-hazards assumption was assessed using scaled Schoenfeld residuals, both for individual covariates and globally. In Model B, the fixed future-surgery variable showed marked non-proportionality (exact *p* = 0.00053; global Model B *p* = 0.00162) [15]. Accordingly, the single hazard ratio from this deliberately time-misaligned fixed-exposure specification was interpreted as a descriptive summary of that coding approach rather than as a stable treatment association over the entire follow-up period. Modest departures from proportional hazards were also observed in Model A (global *p* = 0.045). A fixed-horizon logistic-regression sensitivity analysis was therefore performed among patients with ascertainable 180-day outcome status to evaluate whether the principal prediction findings depended on the proportional-hazards assumption.

### 2.7. Model Performance and Internal Validation

Predictive performance was evaluated at prespecified fixed time horizons using discrimination, overall prediction error, and calibration measures appropriate for prognostic models with time-to-event outcomes [13,16]. Discrimination was quantified using the time-dependent area under the receiver operating characteristic curve (AUC), and overall prediction error was quantified using the Brier score. For the primary analysis, both measures were evaluated at 180 days. Model B was compared directly with Model A using differences in AUC and Brier score, with positive differences in AUC indicating improved discrimination and negative differences in Brier score indicating reduced prediction error.

Apparent performance was first estimated in the complete-case analysis cohort. Internal validation was subsequently performed using 1000-replicate bootstrap cross-validation, with performance evaluated on observations not used for fitting within each bootstrap iteration. The resulting cross-validated AUC and Brier estimates were used to assess out-of-sample performance and the incremental contribution of subsequent surgery. These estimates were treated as internally validated performance measures rather than as classical apparent-minus-test optimism corrections, consistent with contemporary recommendations to evaluate prediction performance beyond apparent model fit [12,13].

Calibration of Model A was assessed separately. Overall calibration was descriptively assessed by comparing mean predicted 180-day risk with the Kaplan–Meier estimate of observed mortality. An optimism-corrected calibration slope was obtained using 1000 bootstrap replicates. A slope of 1 indicates ideal preservation of the prognostic gradient, whereas values below 1 suggest overfitting. Grouped calibration was additionally examined across quintiles of predicted risk. Calibration assessment followed contemporary recommendations to consider both the relative prognostic gradient and agreement between observed and predicted absolute risks rather than relying on discrimination alone [16].

### 2.8. Temporal Validation and Model Transportability

Temporal validation was performed by chronologically separating the complete-case cohort into a derivation cohort comprising patients diagnosed during 2008–2017 and a validation cohort comprising patients diagnosed during 2018–2026. Model coefficients and the baseline hazard were estimated exclusively in the derivation cohort and were applied to the later cohort without refitting or recalibration. Both Model A and Model B were evaluated in this manner.

Temporal discrimination and prediction error were assessed at 180 days using the same AUC and Brier-score framework as in the primary analysis. Calibration of Model A was evaluated by comparing mean predicted risk with Kaplan–Meier observed risk, by grouped calibration across predicted-risk quintiles, and by estimating the calibration slope using the derivation-model linear predictor. Absolute calibration was additionally summarized using the observed-minus-predicted risk difference and the observed-to-expected (O/E) risk ratio, defined as Kaplan–Meier observed mortality divided by mean predicted risk. Uncertainty in these two absolute-calibration measures was quantified using 2000 independent bootstrap resamples of the derivation and validation cohorts, with Model A refitted in each derivation resample and applied without recalibration to the corresponding validation resample. The numbers of 180-day deaths and patients censored alive before 180 days were reported separately for both temporal cohorts.

To explore possible changes in case mix between calendar periods, baseline characteristics and the distribution of the derivation-model prognostic index were compared using standardized mean differences. In an exploratory analysis of residual temporal variation in baseline mortality, the derivation-model prognostic index was retained as an offset in a Cox model containing an indicator for the later calendar period. This analysis was intended to characterize possible temporal baseline-risk drift and was not interpreted as a formal causal era effect.

Complementary full-cohort descriptive analyses summarized microbiological profiles, IE phenotype and structural characteristics according to calendar period, as well as recorded surgical indications and treatment pathways. Variables whose timing relative to the index diagnosis could not be established reliably, including structural complications and surgical indications, were used for descriptive characterization only and were not incorporated into Model A (Supplementary Tables S2–S4).

### 2.9. Sensitivity Analyses

Several sensitivity analyses were undertaken to examine the robustness of the primary findings. First, prediction analyses were repeated at 30-day and 90-day horizons. To preserve temporal alignment between treatment information and the corresponding outcome horizon, Model B incorporated surgery occurring within 30, 90, or 180 days for prediction of mortality at the respective horizon. Apparent performance was evaluated at all three horizons, and bootstrap cross-validation was performed using 1000 replicates. Because only two 30-day deaths occurred among patients undergoing surgery within 30 days, repeated convergence and separation warnings arose during 30-day resampling; these internally validated 30-day estimates were therefore regarded as exploratory.

Second, the functional form of selected baseline predictors was varied by modeling NYHA functional class categorically rather than ordinally and BUN on the log2 scale rather than per 10-mg/dL increase. This analysis assessed whether the principal comparison between Model A and Model B depended materially on the primary coding assumptions.

Third, because proportional-hazards diagnostics identified modest non-proportionality in Model A, a fixed-horizon logistic-regression sensitivity analysis was performed among patients whose 180-day vital status was ascertainable. Patients who died within 180 days or had documented follow-up extending to at least 180 days were included, whereas patients censored alive before 180 days were excluded. Discrimination was assessed using conventional ROC AUCs, paired AUCs were compared using DeLong’s test, and Brier scores and a nested likelihood-ratio test were also calculated.

Fourth, the temporal ambiguity of records in which surgery and death occurred on the same calendar day was examined using three specifications: same-day deaths treated as occurring before surgery, same-day deaths treated as postoperative, and complete exclusion of same-day records. Because precise within-day procedure and death times were unavailable, these alternative specifications were applied both to the overall time-dependent surgery analysis and to the exploratory phase-specific model. For the latter, deaths and accumulated person-time were additionally summarized for the preoperative, postoperative days 0–14, and postoperative >14-day states under each convention.

Additional sensitivity analyses included landmark analyses at days 14 and 30 and flexible time-varying coefficient models to assess whether the surgery–mortality association varied over follow-up. Because landmark analyses condition on survival to the landmark, they address a different estimand from the primary time-dependent analysis; their results are reported in Supplementary Table S5. Flexible time-varying coefficient models were used only as supportive diagnostic analyses; because estimates became unstable in sparsely supported portions of follow-up, individual time-specific estimates were not tabulated or used as principal results.

### 2.10. Missing Data

Primary analyses used complete cases because missingness among the prespecified baseline model predictors was limited. Fifteen of 293 patients (5.1%) had missing information in at least one predictor, predominantly CRP. The extent and pattern of missingness were described, and characteristics and 180-day mortality were compared descriptively between complete and incomplete cases.

As a sensitivity analysis, missing baseline predictors were addressed using multiple imputation by chained equations with 50 imputations. Continuous variables with missing values were imputed using predictive mean matching [17]. The imputation model incorporated the baseline predictor set, the 180-day event indicator, and the Nelson–Aalen cumulative hazard to retain information on the survival outcome [18]. Subsequent surgery was deliberately excluded as an imputation predictor so that post-baseline treatment information could not indirectly enter the baseline predictor set through imputation.

Model A and its fixed-surgery extension were fitted separately within each imputed dataset, and parameter estimates were combined using Rubin’s rules [17]. The nested models with and without subsequent surgery were compared using the D1 multivariate Wald procedure.

### 2.11. Statistical Analysis

Continuous variables were summarized as median with interquartile range or mean with standard deviation, as appropriate, and categorical variables as counts and percentages. Standardized mean differences were used to describe differences between temporal cohorts; absolute SMDs were reported for descriptive assessment of case-mix differences rather than formal hypothesis testing. Because of its skewed distribution, CRP was displayed on the original scale descriptively but entered regression models after log(1 + CRP) transformation.

Cox proportional-hazards models used Efron’s method for tied event times. Time-dependent surgical exposure was constructed using counting-process data with patient follow-up split at the recorded procedure date. Hazard ratios are reported with 95% confidence intervals. All statistical tests were two-sided, and *p* values < 0.05 were considered statistically significant where formal hypothesis testing was undertaken. Because the analyses of postoperative surgery states were observational and susceptible to confounding by indication, statistical significance was not used to infer a causal treatment effect.

Time-dependent AUCs, Brier scores, and bootstrap prediction validation were performed using the riskRegression framework; survival models and time-dependent covariates were implemented using the survival package. Multiple imputation was performed using mice, and ROC analyses for the fixed-horizon logistic sensitivity analysis used pROC. Analyses were conducted in R (R Foundation for Statistical Computing, Vienna, Austria, version 4.6.0).

## 3. Results

### 3.1. Study Population and Outcomes

A total of 293 patients with infective endocarditis were included in the study (Figure 1). The median age was 57.0 years (IQR, 44.0–69.0), and 179 patients (61.1%) were male. Coronary artery disease was present in 25.8%, heart failure in 18.1%, chronic kidney disease in 27.6%, and dialysis dependence in 22.9% of patients. At presentation, 22.9% were in NYHA functional class III or IV. Median hemoglobin was 10.5 g/dL (IQR, 9.4–12.1), median blood urea nitrogen was 22.0 mg/dL (IQR, 15.0–36.0), median C-reactive protein was 102.0 mg/L (IQR, 51.8–171.2), and median left ventricular ejection fraction was 60.0% (IQR, 50.0–60.0). Severe valvular regurgitation was present in 129 patients (44.0%), and 110 cases (37.5%) were blood-culture negative (Table 1).

During follow-up, 122 patients (41.6%) underwent surgery within 180 days of the index date. Overall, 130 patients underwent surgery during follow-up, including 122 within 180 days and 8 thereafter. Treatment pathways, recorded reasons for recommended but unperformed surgery, and surgical indications are detailed in Supplementary Table S4. All-cause mortality occurred in 34 patients (11.6%) by 30 days, 72 (24.6%) by 90 days, and 92 (31.4%) by 180 days. Fifteen patients (5.1%) had missing data for at least one prespecified baseline model predictor, predominantly C-reactive protein, leaving 278 patients in the primary complete-case modeling cohort. This cohort was chronologically divided into a derivation cohort from 2008–2017 (*n* = 155) and a temporal validation cohort from 2018–2026 (*n* = 123) (Figure 1).

Comparison of the two calendar periods showed evidence of temporal case-mix variation rather than a uniform shift toward greater measured baseline severity. Coronary artery disease was more frequent in the later period (34.9% vs. 18.8%; absolute standardized mean difference [SMD], 0.370), whereas log-transformed C-reactive protein was lower (absolute SMD, 0.267). Smaller differences were observed for age (SMD, 0.169), heart failure (SMD, 0.115), and blood urea nitrogen (SMD, 0.105), while sex, chronic kidney disease, dialysis, severe regurgitation, and blood-culture-negative status were broadly similar between periods (Table 1).

### 3.2. Baseline Prediction of 180-Day Mortality

**Figure 2:**
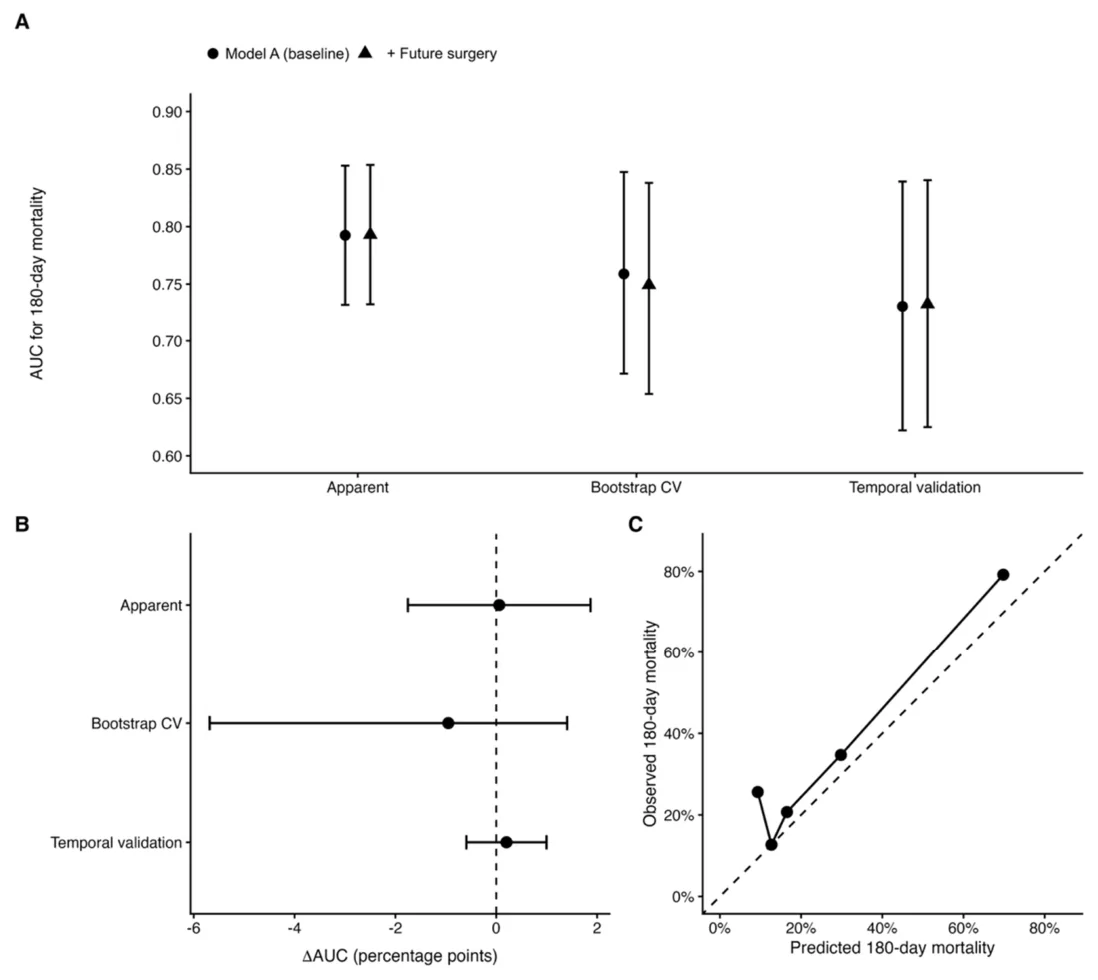
Predictive performance and temporal validation of the 180-day mortality models. (**A**) Discrimination of the baseline model (Model A) and the model additionally incorporating subsequent surgery within 180 days (Model B) across apparent analysis, 1000-replicate bootstrap cross-validation, and temporal validation. Points represent area under the receiver operating characteristic curve (AUC) estimates and error bars indicate 95% confidence intervals. (**B**) Incremental discrimination associated with the addition of subsequent surgery, expressed as the difference in AUC between Model B and Model A. The dashed vertical line denotes no incremental discrimination. (**C**) Grouped calibration of Model A in the temporally separated 2018–2026 validation cohort. The dashed diagonal line represents perfect calibration. The temporal calibration slope was 1.025 (95% CI, 0.711–1.338), while mean predicted 180-day mortality was 27.3% compared with an observed Kaplan–Meier risk of 34.7% (95% CI, 25.5–42.8%). The observed-minus-predicted difference was +7.5 percentage points (bootstrap 95% CI, −2.4 to +18.8), indicating a tendency toward absolute-risk underprediction with substantial uncertainty.

Model A, using the seven prespecified predictors obtained during the initial index-episode evaluation, showed an apparent AUC of 0.792 (95% CI, 0.732–0.853) for 180-day mortality (Table 2; Figure 2A). The apparent Brier score was 0.163. At 180 days, mean predicted mortality closely matched the Kaplan–Meier estimate of observed risk (32.6% vs. 32.7%). After 1000-replicate bootstrap cross-validation, discrimination was attenuated but remained moderate, with a cross-validated AUC of 0.759 (95% CI, 0.671–0.847) and a Brier score of 0.180. The optimism-corrected calibration slope was 0.907, indicating modest overfitting but preservation of the overall prognostic gradient (Table 2). Full model coefficients and the 180-day baseline survival function are provided in Supplementary Table S1 to permit independent reconstruction of predicted risks.

Proportional-hazards diagnostics suggested modest departures from proportionality for NYHA functional class and log-transformed CRP, with a borderline global test for Model A (*p* = 0.045; Supplementary Table S6). The principal prediction comparison was therefore also examined using fixed-horizon logistic regression among 261 patients with ascertainable 180-day status. Model A and Model B both yielded an AUC of 0.812; the AUC difference was +0.00039 (95% CI, −0.00601 to +0.00523), and addition of surgery did not improve model fit (likelihood-ratio *p* = 0.655) (Supplementary Table S7). This analysis also excluded the 17 patients censored alive before day 180, for whom future surgery status through the full prediction horizon could not be established with certainty.

### 3.3. Incremental Predictive Value of Subsequent Surgery

Adding the binary indicator of surgery within 180 days to the seven-predictor Model A produced no detectable improvement in apparent predictive performance. The AUC was 0.793 (95% CI, 0.732–0.854), compared with 0.792 for Model A, corresponding to an AUC difference of only 0.001 (95% CI, −0.018 to 0.019; *p* = 0.949) (Table 2; Figure 2A,B). Prediction error was similarly unchanged (Brier score, 0.164 vs. 0.163).

No improvement in discrimination was detected after internal validation. In 1000-replicate bootstrap cross-validation, Model B had an AUC of 0.749 (95% CI, 0.654–0.838), compared with 0.759 (95% CI, 0.671–0.847) for Model A. The cross-validated difference in AUC was −0.010 (95% CI, −0.057 to 0.014), while the corresponding Brier scores were 0.182 and 0.180, respectively. Thus, within this seven-predictor modeling framework, adding future surgery did not detectably improve out-of-sample prediction of 180-day mortality.

A similar pattern was observed across the prespecified prediction horizons. When surgery was defined separately within each corresponding horizon, apparent differences in AUC between Models B and A were +0.72 percentage points at 30 days, +0.29 percentage points at 90 days, and +0.06 percentage points at 180 days, with all confidence intervals including zero (Supplementary Table S8). Bootstrap cross-validation likewise showed no evidence of incremental discrimination at 90 or 180 days. Thirty-day bootstrap estimates were considered exploratory because only two deaths occurred within 30 days among patients undergoing surgery within the same horizon, resulting in repeated convergence warnings during resampling.

### 3.4. Temporal Validation and Calibration

When Model A was fitted exclusively in the 2008–2017 complete-case derivation cohort (*n* = 155; 47 deaths by 180 days) and applied without refitting to the 2018–2026 complete-case validation cohort (*n* = 123; 41 deaths by 180 days), discrimination remained moderate, with an AUC of 0.731 (95% CI, 0.622–0.839) and a Brier score of 0.177 (Table 2; Figure 2A). Addition of subsequent surgery again produced no detectable improvement in discrimination in the temporal validation cohort: Model B yielded an AUC of 0.733 (95% CI, 0.625–0.840), corresponding to an AUC difference of +0.002 (95% CI, −0.006 to +0.010), while Brier scores were 0.176 and 0.177 for Models B and A, respectively (Figure 2B).

Eight patients in the derivation cohort and nine in the validation cohort were censored alive before day 180. The temporal calibration slope was 1.025 (95% CI, 0.711–1.338), indicating substantial uncertainty around preservation of the prognostic gradient. Absolute calibration showed a tendency toward underprediction: mean predicted 180-day mortality was 27.3%, whereas the observed Kaplan–Meier risk was 34.7%. The observed-minus-predicted difference was +7.5 percentage points, with a bootstrap 95% CI of −2.4 to +18.8 percentage points. The corresponding observed-to-expected risk ratio was 1.27 (bootstrap 95% CI, 0.93–1.88). Thus, the later cohort showed possible absolute-risk underprediction, although the uncertainty around the calibration difference was substantial. Grouped calibration showed a similar tendency toward underprediction at the lower and upper ends of the predicted-risk distribution.

Temporal case-mix analyses did not indicate a uniformly more adverse measured risk profile in the validation cohort. The mean prognostic index derived from Model A was slightly lower in the later cohort (standardized mean difference, 0.137), despite its higher observed 180-day mortality. In a model retaining the derivation prognostic index as an offset, the later calendar period was associated with a hazard ratio of 1.47 (95% CI, 0.97–2.24; *p* = 0.070) (Supplementary Table S9). These findings were compatible with temporal baseline-risk drift, although the estimate did not provide definitive evidence for an independent era effect. In complementary full-cohort descriptive analyses, the later period included a higher proportion of prosthetic-valve IE (16.7% vs. 9.6%) and oncological comorbidity (7.9% vs. 2.4%). In contrast, septic shock (24.6% vs. 24.6%), blood-culture negativity (38.1% vs. 37.1%), and maximum vegetation size (median, 15 mm in both periods) were broadly similar. Recorded S. aureus proportions were 5.6% in 2018–2026 and 7.2% in 2008–2017; however, seven culture-positive episodes in the later period lacked organism-level specification, limiting organism-specific temporal comparisons (Supplementary Tables S2 and S3).

### 3.5. Impact of Temporal Exposure Definition on the Surgery–Mortality Association

Although adding future surgery to Model A produced no detectable predictive improvement, the estimated surgery–mortality association changed substantially according to its temporal representation. In the full complete-case cohort (*n* = 278), treating eventual surgery within 180 days as a fixed baseline covariate yielded an adjusted HR of 0.71 (95% CI, 0.44–1.17; *p* = 0.181). Proportional-hazards diagnostics demonstrated marked non-proportionality for this fixed future-surgery variable (exact *p* = 0.00053; global Model B *p* = 0.00162), indicating that a single fixed-exposure HR should not be interpreted as a stable association throughout follow-up.

**Figure 3:**
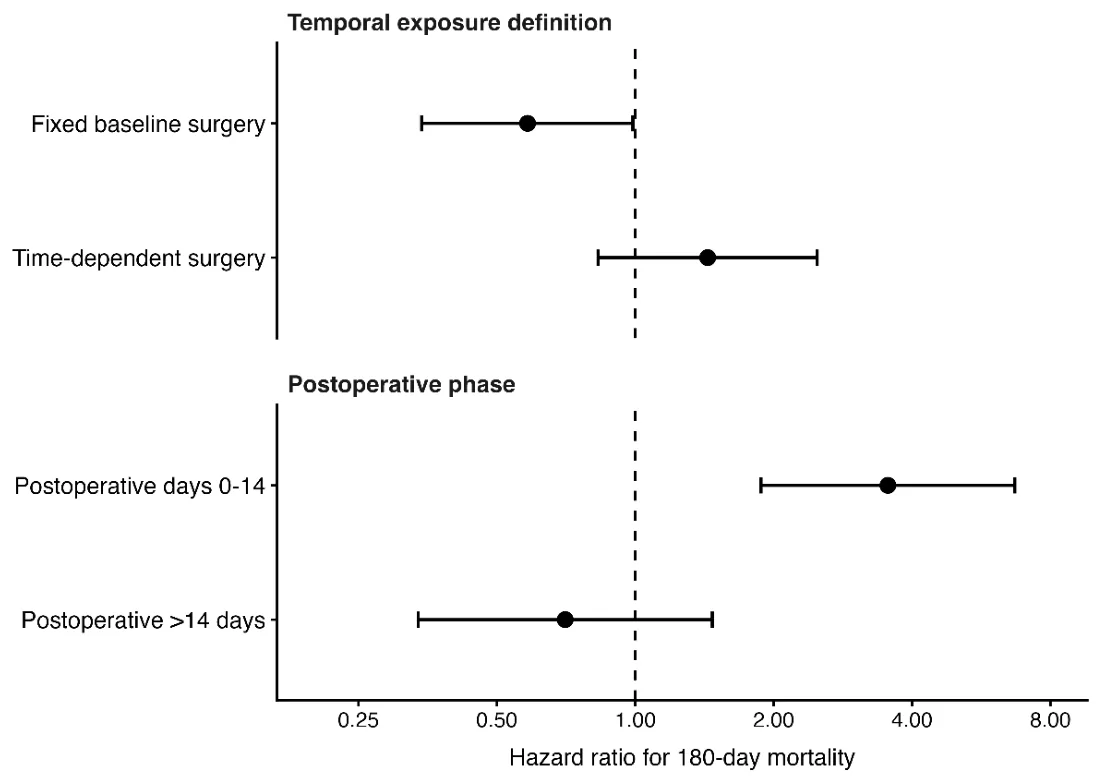
Association of surgery with 180-day mortality according to temporal exposure definition. Adjusted hazard ratios are shown for surgery coded as a fixed baseline exposure, surgery modeled as a time-dependent exposure according to the actual procedure date, and postoperative phase-specific states. Five patients in whom surgery and death were recorded on the same calendar day were excluded because within-day temporal ordering could not be determined. Models were adjusted for age, NYHA functional class, hemoglobin, blood urea nitrogen, left ventricular ejection fraction, severe regurgitation, and log-transformed C-reactive protein. Phase-specific estimates compare the postoperative 0–14-day and >14-day states with the not-yet-operated state. The dashed vertical line denotes a hazard ratio of 1.0. The hazard-ratio axis is displayed on a logarithmic scale. These estimates represent observational associations and should not be interpreted as causal effects of surgery, because time-dependent modeling addresses immortal-time allocation but does not eliminate confounding by indication.

For the direct fixed-versus-time-dependent comparison, five patients with surgery and death recorded on the same calendar day were excluded because within-day ordering was unavailable. In the resulting matched sample of 273 patients, fixed baseline coding yielded an adjusted HR of 0.58 (95% CI, 0.34–0.99), whereas updating surgical status at the actual procedure date yielded an adjusted HR of 1.44 (95% CI, 0.83–2.49) (Table 3; Figure 3). The magnitude and nominal statistical significance of the fixed-exposure estimate were therefore sensitive to sample definition. More importantly, the substantial change in the point estimate after incorporating actual treatment timing demonstrates sensitivity of the surgery–mortality association to temporal exposure coding; it should not be interpreted as an estimate of the benefit or harm of surgery. Thus, the lower hazard estimate produced by fixed baseline coding was not reproduced when the post-baseline timing of surgery was incorporated into the exposure definition.

The magnitude of the time-dependent estimate was sensitive to the handling of five same-day surgery/death records because within-day ordering was unavailable. Classifying these deaths as occurring before surgery yielded an adjusted HR of 1.29 (95% CI, 0.75–2.20), whereas classifying them as postoperative yielded an HR of 1.84 (95% CI, 1.10–3.09); excluding the five records yielded the primary same-sample estimate of 1.44 (95% CI, 0.83–2.49) (Supplementary Table S10). This sensitivity to day-level event ordering further supported avoiding interpretation of any single time-dependent estimate as a causal effect of surgery.

### 3.6. Postoperative Phase-Specific Mortality Association

Exploratory phase-specific analyses suggested a higher mortality hazard during the first 14 postoperative days relative to the not-yet-operated state. Under the primary convention excluding the five same-day surgery/death records, the adjusted HR was 3.54 (95% CI, 1.88–6.69) for postoperative days 0–14 and 0.70 (95% CI, 0.34–1.47) thereafter (Table 3; Figure 3). The magnitude of the early postoperative association was sensitive to same-day event handling: when same-day deaths were classified as preoperative, postoperative, or excluded, the corresponding early postoperative HRs were 3.19, 5.00, and 3.54, whereas estimates beyond day 14 remained more stable at 0.64, 0.71, and 0.70, respectively (Supplementary Table S10). Under the exclusion convention, 60 deaths occurred during 22,900.5 preoperative person-days, 13 during 1476.0 person-days in postoperative days 0–14, and 10 during 11,922.0 person-days thereafter. These phase-specific analyses were exploratory and should not be interpreted as causal effects of surgery.

### 3.7. Sensitivity and Robustness Analyses

Landmark analyses are reported in Supplementary Table S5. Flexible time-varying coefficient models were used only as supportive diagnostic analyses; because estimates became unstable in sparsely supported portions of follow-up, individual time-specific estimates were not tabulated or used as principal results. The principal prediction findings were also robust to alternative model specifications. Replacing ordinal NYHA and linearly scaled BUN with categorical NYHA and log2-transformed BUN produced virtually unchanged discrimination and again showed no material benefit from adding subsequent surgery. In a fixed-horizon logistic-regression sensitivity analysis restricted to 261 patients with ascertainable 180-day status, the AUCs were 0.812 for both Model A and Model B; the difference in AUC was 0.0004 (95% CI, −0.0060 to +0.0052; DeLong *p* = 0.891), and addition of surgery did not improve model fit (likelihood-ratio *p* = 0.655) (Supplementary Table S7).

Among the 68 patients with CIED-related IE, all underwent device-system extraction after the index date. Thirty-seven had a recorded surgical procedure date and underwent surgical extraction; the median time from index date to surgery was 21 days (IQR, 11–43; range, 2–882 days). Thirty-four of these procedures occurred within 180 days and three thereafter. The remaining 31 patients underwent extraction without a recorded cardiac surgical procedure and were therefore not classified as surgically treated under the primary cardiac-surgery exposure definition. Exact extraction dates for the 31 procedures without a recorded cardiac surgical intervention were not retained as a separate structured field. Among the 37 procedures recorded as surgical interventions, the retrospective dataset did not reliably distinguish surgical extraction alone from extraction performed concomitantly with another cardiac surgical procedure.

The principal fixed-versus-time-dependent comparison was also preserved after exclusion of CIED-related IE. Among 211 non-CIED patients in the matched analysis, fixed baseline coding yielded an adjusted HR of 0.50 (95% CI, 0.27–0.93), whereas the corresponding time-dependent estimate was 1.43 (95% CI, 0.77–2.66) (Supplementary Table S11).

Missing baseline data were limited to 15 patients (5.1%), predominantly because of missing C-reactive protein values. Multiple imputation with 50 imputations yielded results consistent with the complete-case analyses. In the fixed-surgery model, the pooled HR for subsequent surgery was 0.67 (95% CI, 0.41–1.11; *p* = 0.116), and adding subsequent surgery to Model A did not significantly improve model fit (D1 *p* = 0.116) (Supplementary Table S12).

## 4. Discussion

In this longitudinal cohort of patients with infective endocarditis, the principal finding was a divergence between the predictive contribution of subsequent surgery and the association estimates obtained according to its temporal representation. Within the prespecified seven-predictor framework, adding a binary indicator of subsequent surgery produced no detectable improvement in discrimination or prediction error in apparent analysis, bootstrap cross-validation, or temporal validation. In contrast, the estimated surgery–mortality association changed substantially when eventual surgery was represented according to its actual timing rather than assigned retrospectively from baseline. Thus, in this cohort and modeling framework, the temporal coding of subsequent treatment had a much larger impact on association estimates than on predictive performance. Exploratory postoperative phase analyses additionally suggested temporal heterogeneity, but the magnitude of the early postoperative association varied according to the handling of same-day surgery/death records and should not be interpreted as a treatment effect.

Contemporary IE prediction research has also moved toward models explicitly anchored to the initial clinical evaluation. The recently reported EURO-ENDO score was developed in a large prospective European cohort and externally validated in a temporally independent cohort using variables available during the initial diagnostic assessment. Although its target population and 30-day endpoint differ from those of the present study, it illustrates the current emphasis on temporal alignment between predictor availability and the intended prediction time [3].

Our findings are particularly relevant in light of recent efforts to develop machine-learning models for intermediate-term mortality in IE. Wu et al. recently developed a 6-month mortality model in 444 patients in which surgical intervention was one of nine selected predictors and emerged as the feature most strongly associated with lower predicted risk [5]. Their observed 6-month mortality was markedly lower among surgically treated patients, yet the authors explicitly acknowledged that this association reflected treatment selection, confounding by indication, and immortal-time bias rather than an isolated treatment effect [5]. Importantly, exclusion of surgery from their model left a C-index of 0.804 and did not significantly impair overall model performance [5]. Our results extend this observation by explicitly evaluating the same conceptual issue from the prediction-time perspective: when subsequent surgery was deliberately added to an otherwise identical baseline model, the increase in apparent AUC was approximately zero and remained absent after bootstrap and temporal validation. In our cohort, therefore, the strong outcome association of subsequent surgery did not translate into detectable incremental predictive improvement when it was added as a binary future-treatment indicator to the prespecified seven-predictor model.

The more pronounced consequence of surgery coding in our cohort concerned association rather than prediction. In all 278 complete cases, the fixed future-surgery specification yielded an adjusted HR of 0.71 (95% CI, 0.44–1.17), whereas after exclusion of the five same-day surgery/death records, the corresponding estimate was 0.58 (95% CI, 0.34–0.99). Moreover, the fixed future-surgery variable showed marked non-proportionality (exact *p* = 0.00053), indicating that neither fixed estimate should be interpreted as a stable association throughout follow-up. In the matched 273-patient sample, updating exposure at the actual procedure date yielded an adjusted HR of 1.44 (95% CI, 0.83–2.49). This contrast demonstrates the importance of temporal exposure coding but does not estimate the causal benefit or harm of surgery. This pattern is consistent with the established structure of immortal-time bias: patients ultimately classified as treated must survive long enough to receive treatment, and assigning their pre-treatment survival time to the treated state can create an artificial survival advantage. This problem has recently been highlighted specifically in studies of cardiac surgery for IE, and contemporary methodological guidance emphasizes alignment of eligibility, treatment assignment, and time zero when analyzing post-baseline interventions [9,14].

The time-dependent analysis should not, however, be interpreted as estimating the causal effect of surgery. Surgery in IE is selected according to clinical indications, operative feasibility, neurological status, infection control, haemodynamic compromise, comorbidity, and perceived procedural risk. Contemporary guidelines recommend surgery for defined clinical scenarios including heart failure, uncontrolled infection, and prevention of embolic complications, meaning that surgical treatment is intrinsically linked to disease severity and clinical decision-making [1]. Contemporary nationwide data further show that a substantial proportion of patients meeting surgical indications do not ultimately undergo surgery and that treated and untreated groups differ markedly in age and clinical characteristics, underscoring the potential for confounding by indication in observational comparisons [10]. Time-dependent modeling corrects the temporal allocation of exposure but does not eliminate confounding by indication [14,19]. This limitation is particularly important for the exploratory phase-specific analysis. Although the direction of the early postoperative association was preserved across alternative same-day conventions, its magnitude varied appreciably, with adjusted HRs ranging from 3.19 to 5.00. The early postoperative state may capture perioperative risk, operative urgency, confounding by indication, residual infection, and other evolving severity factors not represented by the baseline adjustment set. Conversely, patients surviving beyond the immediate postoperative period constitute a selected population. The 14-day division was therefore used only to characterize temporal heterogeneity and should not be interpreted as a validated biological threshold or as evidence that surgery itself increases early mortality. Complementary landmark analyses addressed a different conditional estimand and were not considered confirmatory.

Another important finding was the distinction between relative and absolute transportability over calendar time. When the model developed in 2008–2017 was applied without refitting to patients treated during 2018–2026, discrimination remained moderate, while the calibration slope was imprecisely estimated at 1.025 (95% CI, 0.711–1.338). Mean predicted 180-day mortality was 27.3% compared with an observed risk of 34.7%, corresponding to an observed-minus-predicted difference of +7.5 percentage points (bootstrap 95% CI, −2.4 to +18.8) and an O/E risk ratio of 1.27 (bootstrap 95% CI, 0.93–1.88). These estimates suggest possible underprediction in the later cohort but do not establish systematic miscalibration. This discrepancy was not explained by a uniformly more adverse measured baseline profile, as the derivation-model prognostic index was not higher in the validation cohort. The extended descriptive case-mix assessment provided a similar picture: although prosthetic-valve IE and oncological comorbidity were more frequent in the later period, blood-culture negativity, septic shock, and maximum vegetation size were broadly comparable across eras. Organism-specific temporal comparisons were less certain because seven culture-positive episodes in the later period lacked organism-level specification (Supplementary Tables S2 and S3). IE populations are known to evolve over time with changes in age, cardiovascular comorbidity, microbiology, device exposure, referral patterns, and treatment strategies [20]. A large contemporary analysis of 86,469 cases documented substantial temporal shifts in patient characteristics and causative microorganisms [20]. Our finding is therefore compatible with baseline-risk drift or unmeasured case-mix change, although the present data cannot distinguish these mechanisms. From a prediction perspective, this emphasizes that preservation of discrimination or calibration slope alone does not guarantee accurate absolute-risk estimation in a later clinical era.

Several features strengthen the present study. The index date was explicitly anchored to the initial clinical recognition of IE, and baseline/index-episode information was separated conceptually from subsequent treatment information. The incremental value of subsequent surgery was evaluated against an otherwise identical baseline model rather than inferred from variable importance alone. Internal validation used bootstrap cross-validation rather than a small random holdout sample, and model transportability was additionally assessed in a chronologically separated cohort. The surgery analyses compared fixed and time-dependent exposure definitions in the same patients with the same adjustment set, thereby isolating the influence of temporal exposure coding. Multiple sensitivity analyses addressed temporal ambiguity, model specification, missing data, and non-proportionality.

The study also has important limitations. First, its retrospective, single-center design and relatively modest sample size limit precision and generalizability, particularly for phase-specific and subgroup analyses. Temporal validation within the same institution provides a stronger test than apparent performance alone but does not substitute for external validation in geographically and clinically independent populations. The relatively small temporal validation cohort, with 41 deaths within 180 days, also resulted in substantial uncertainty around calibration estimates, including both the calibration slope and the magnitude of the observed–predicted calibration difference. Second, Model A was intentionally parsimonious and was constructed as a methodological reference model rather than as a comprehensive clinical risk score. It did not include all potentially relevant baseline prognostic characteristics, including septic shock and dialysis dependence, nor did it capture detailed microbiology, anatomic surgical indications, neurological status, operative urgency, frailty, or subsequent time-varying complications. Accordingly, the present findings should not be interpreted as demonstrating that subsequent surgery would lack incremental predictive value when added to every possible baseline IE risk model. Although broad microbiological profiles were descriptively examined, organism-level information was unavailable for seven culture-positive cases in the later period, limiting detailed temporal comparisons. Third, procedure and death times were available at calendar-day rather than within-day resolution; the sensitivity of estimates to the five same-day records demonstrates the importance of this limitation. Fourth, the 14-day postoperative division should be viewed as an exploratory characterization of temporal heterogeneity rather than a validated biological threshold. Fifth, missing baseline data were limited but were handled primarily through complete-case analysis, although multiple-imputation sensitivity analyses produced similar findings. Finally, Model B intentionally incorporated information unavailable at diagnosis and should not be interpreted as a clinically deployable prediction model; it served as a methodological comparator to evaluate the consequences of introducing future treatment information into a baseline prediction framework intended for use during the initial index-episode evaluation. In addition, reliable national vital-status linkage was unavailable for 17 foreign-national patients, who were therefore censored at their last documented clinical follow-up; however, fixed-horizon sensitivity analyses excluding patients without ascertainable 180-day status yielded consistent predictive conclusions. Although the present cohort was reconstructed from a new review of institutional records, it partially overlaps with a previously reported single-center cohort; exact overlap with that study’s final analytic subset could not be reconstructed from the archived screening records.

## 5. Conclusions

In this cohort of patients with infective endocarditis, adding a binary indicator of subsequent surgery to the prespecified seven-predictor baseline model produced no detectable improvement in 180-day mortality prediction. This finding should not be generalized to all possible baseline risk models or interpreted as indicating that surgery carries no prognostic information. In contrast, the estimated surgery–mortality association changed substantially according to whether future surgery was coded as a fixed baseline characteristic or updated according to its actual timing. These findings emphasize the distinction between prediction and treatment–outcome association: prediction models should respect the intended prediction time, while observational analyses of post-baseline treatments should explicitly account for treatment timing.

## Figures and Tables

**Table 1:** Baseline characteristics, treatment, and outcomes according to calendar period.

Characteristic	Overall (*n* = 293)	2008–2017 (*n* = 167)	2018–2026 (*n* = 126)	SMD
**Demographics and comorbidities**				
Age, years	57.0 [44.0–69.0]	57.0 [41.0–69.0]	58.0 [47.0–69.5]	0.169
Male sex	179 (61.1%)	102 (61.1%)	77 (61.1%)	0.001
Coronary artery disease	75 (25.8%)	31 (18.8%)	44 (34.9%)	0.370
Heart failure	53 (18.1%)	27 (16.2%)	26 (20.6%)	0.115
Chronic kidney disease	81 (27.6%)	47 (28.1%)	34 (27.0%)	0.026
Dialysis	67 (22.9%)	40 (24.0%)	27 (21.4%)	0.060
**IE phenotype**				
Native-valve IE	175 (59.7%)	107 (64.1%)	68 (54.0%)	0.207
Prosthetic-valve IE	37 (12.6%)	16 (9.6%)	21 (16.7%)	0.211
CIED-related IE	68 (23.2%)	37 (22.2%)	31 (24.6%)	0.058
Mixed/combined IE	10 (3.4%)	5 (3.0%)	5 (4.0%)	0.053
**Clinical and laboratory characteristics**				
NYHA class I	171 (58.4%)	95 (56.9%)	76 (60.3%)	—
NYHA class II	55 (18.8%)	31 (18.6%)	24 (19.0%)	—
NYHA class III	27 (9.2%)	12 (7.2%)	15 (11.9%)	—
NYHA class IV	40 (13.7%)	29 (17.4%)	11 (8.7%)	—
Hemoglobin, g/dL	10.5 [9.4–12.1]	10.5 [9.3–12.1]	10.4 [9.4–12.0]	0.057
BUN, mg/dL	22.0 [15.0–36.0]	22.5 [15.0–36.0]	21.0 [16.0–36.0]	0.105
CRP, mg/L	102.0 [51.8–171.2]	103.0 [59.0–175.0]	98.0 [36.5–165.0]	0.267
LVEF, %	60.0 [50.0–60.0]	60.0 [50.0–60.0]	60.0 [55.0–60.0]	0.039
Severe regurgitation	129 (44.0%)	70 (41.9%)	59 (46.8%)	0.099
Blood-culture-negative infective endocarditis	110 (37.5%)	62 (37.1%)	48 (38.1%)	0.020
**Treatment**				
Surgery within 180 days	122 (41.6%)	72 (43.1%)	50 (39.7%)	—
Outcomes				
30-day mortality	34 (11.6%)	18 (10.8%)	16 (12.7%)	—
90-day mortality	72 (24.6%)	37 (22.2%)	35 (27.8%)	—
180-day mortality	92 (31.4%)	49 (29.3%)	43 (34.1%)	—

**Footnote:** Continuous variables are presented as median [interquartile range] and categorical variables as n (%). Percentages are based on available observations. Coronary artery disease was missing in 2 patients, BUN in 1, LVEF in 1, and CRP in 13. Standardized mean differences (SMDs) are presented as absolute values. CRP is shown on its original mg/L scale, whereas its SMD was calculated using log-transformed CRP because of its skewed distribution. Treatment and outcome variables are not baseline case-mix characteristics and therefore SMDs are not shown. IE phenotype was unknown in 3 patients. CIED denotes a cardiac implantable electronic device.

**Table 2:** Performance of the 180-day mortality prediction models.

**Panel A. Discrimination and Prediction Error**						
**Evaluation**	**Model A AUC (95% CI)**	**Model B AUC (95% CI)**	**ΔAUC, B − A (95% CI)**	**Model A Brier**	**Model B Brier**	**ΔBrier, B − A**
Apparent performance	0.792 (0.732–0.853)	0.793 (0.732–0.854)	+0.001 (−0.018 to +0.019)	0.163	0.164	+0.0003
1000-replicate bootstrap cross-validation	0.759 (0.671–0.847)	0.749 (0.654–0.838)	−0.010 (−0.057 to +0.014)	0.180	0.182	+0.0025 (−0.0032 to 0.0164)
Temporal validation, 2018–2026	0.731 (0.622–0.839)	0.733 (0.625–0.840)	+0.002 (−0.006 to +0.010)	0.177	0.176	−0.0007 (−0.0018 to 0.0005)
**Panel B. Calibration of Model A**						
**Evaluation**	**Observed 180-Day Risk**	**Mean Predicted Risk**	**Observed − Predicted Differenc**e	**O/E Risk Ratio**		**Calibration Slope**
Full analysis cohort, apparent	32.7%	32.6%				1.000
Bootstrap internal validation	—	—				0.907, optimism-corrected
Temporal validation, 2018–2026	34.7% (95% CI 25.5–42.8)	27.3%	+7.5 pp (bootstrap 95% CI, −2.4 to +18.8)	1.27 (bootstrap 95% CI, 0.93–1.88)		1.025 (95% CI 0.711–1.338)

**Footnote:** Model A included the seven prespecified predictors obtained during the initial index-episode evaluation. Model B additionally incorporated subsequent surgery within 180 days. AUC differences are Model B minus Model A. The observed temporal-validation risk was estimated by Kaplan–Meier analysis. Confidence intervals for the absolute observed-minus-predicted difference and observed-to-expected risk ratio were obtained by bootstrap resampling as described in the Methods.

**Table 3:** Association of surgery with 180-day mortality according to temporal exposure definition.

**Panel A. Fixed Baseline Versus Time-Dependent Coding**			
**Exposure Definition**	***n***	**HR (95% CI)**	***p* Value**
**Full complete-case analysis**			
Adjusted fixed baseline surgery	278	0.71 (0.44–1.17)	0.181
**Matched analysis excluding same-day surgery/death records**			
Crude fixed baseline surgery	273	0.44 (0.27–0.72)	<0.001
Crude time-dependent surgery	273	0.93 (0.56–1.55)	0.787
Adjusted fixed baseline surgery	273	0.58 (0.34–0.99)	0.045
Adjusted time-dependent surgery	273	1.44 (0.83–2.49)	0.196
**Panel B. Postoperative Phase-Specific Associations**			
**Temporal State**	**HR (95% CI)**		***p* Value**
Pre-surgery	1.00 (reference)		—
Postoperative days 0–14	3.54 (1.88–6.69)		<0.001
Postoperative >14 days	0.70 (0.34–1.47)		0.351
**Formal contrast**	HR ratio (95% CI)		*p* value
Postoperative days 0–14 vs. postoperative >14 days	5.03 (2.09–12.14)		<0.001

**Footnote:** The full complete-case fixed-exposure estimate included all 278 patients. For the direct fixed-versus-time-dependent comparison and phase-specific analyses, five patients with surgery and death recorded on the same calendar day were excluded because within-day ordering was unavailable, leaving 273 patients. Adjusted models included age, NYHA functional class, hemoglobin, BUN, LVEF, severe regurgitation, and log-transformed CRP. The fixed future-surgery variable showed marked non-proportionality (exact Schoenfeld-residual *p* = 0.00053); its HR should therefore be interpreted as a summary of a deliberately time-misaligned exposure specification rather than as a stable treatment association. All surgery-related estimates are observational and should not be interpreted as causal effects of surgery.

## Data Availability

The datasets generated and/or analyzed during the current study are not publicly available because they contain potentially sensitive clinical information but may be available from the corresponding author on reasonable request, subject to institutional and ethical approval.

## Supplementary Materials

The following supporting information can be downloaded at: https://www.mdpi.com/article/10.3390/diagnostics16193209/s1. Table S1: Coefficients and baseline survival function of the primary seven-predictor Model A; Table S2: Microbiological characteristics according to calendar period; Table S3: Infective endocarditis phenotype and structural characteristics according to calendar period; Table S4: Surgical indications and treatment pathways; Table S5: Landmark analyses of surgery and subsequent 180-day mortality; Table S6: Proportional-hazards diagnostics for the primary Cox models; Table S7: Fixed-horizon 180-day logistic-regression sensitivity analysis; Table S8: Horizon-specific predictive performance of Model A and Model B; Table S9: Temporal case-mix and calibration diagnostics; Table S10: Sensitivity of time-dependent and phase-specific surgery estimates to same-day surgery/death handling; Table S11: Sensitivity analysis excluding patients with CIED-related infective endocarditis; Table S12: Multiple-imputation sensitivity analysis for missing baseline predictors.
