# Supplementary material for: Incremental Predictive Value of Post-Baseline Surgery and the Impact of Temporal Coding on Mortality Associations in Infective Endocarditis: A Temporally Validated Cohort Study
Source: Diagnostics (Basel). 2026 Oct 2;16(19):3209. doi: 10.3390/diagnostics16193209 (PMC13664497; doi:10.3390/diagnostics16193209)
Supplement: Supplementary file 1 [file diagnostics-16-03209-s001.zip › diagnostics-4593892-supplementary.pdf]

Supplementary Table S1. Coefficients and baseline survival function of the primary seven-predictor Model A

Predictor	Model coding	β coefficient	SE	HR (95% CI)	p value
Age	per 10-year increase	0.1174	0.0732	1.125 (0.974–1.298)	0.109
NYHA functional class	per 1-class increase	0.7829	0.0914	2.188 (1.829–2.617)	<0.001
Hemoglobin	per 1 g/dL increase	0.0368	0.0553	1.038 (0.931–1.156)	0.505
BUN	per 10 mg/dL increase	0.0657	0.0389	1.068 (0.990–1.152)	0.091
LVEF	per 5-percentage-point increase	0.0233	0.0520	1.024 (0.924–1.133)	0.654
Severe regurgitation	yes vs no	0.3188	0.2271	1.375 (0.881–2.147)	0.160
log(1+CRP)	per 1-unit increase	0.1248	0.1173	1.133 (0.900–1.426)	0.287

Uncentered baseline cumulative hazard at 180 days, $H_0(180)$: 0.00878585

Uncentered baseline survival at 180 days, $S_0(180)$: 0.99125264

Footnote: Model A was fitted in the complete-case cohort (n=278; 88 deaths by 180 days) using Efron's method for tied event times. Age was modeled per 10-year increase, BUN per 10-mg/dL increase, LVEF per 5-percentage-point increase, NYHA functional class as an ordinal variable, severe regurgitation as a binary indicator, and CRP as log(1+CRP). The reported baseline survival is the uncentered Cox baseline survival corresponding to all model covariates equal to zero. Individual 180-day mortality risk can be reproduced as:

$Risk_{180} = 1 - S_0(180)$^exp(LP),

where

LP = 0.117431(age/10) + 0.782892(NYHA) + 0.036828(hemoglobin) + 0.065687(BUN/10) + 0.023288(LVEF/5) + 0.318794(severe regurgitation) + 0.124804·log(1+CRP).

Using these coefficients and $S_0(180)$=0.99125264 reproduced the mean predicted 180-day mortality in the complete-case cohort (32.6%).

Supplementary Table S2. Microbiological characteristics according to calendar period

Microbiological characteristic	Overall (N=293)	2008–2017 (N=167)	2018–2026 (N=126)	Absolute SMD
Blood-culture negative	110 (37.5%)	62 (37.1%)	48 (38.1%)	0.020
Blood-culture positive	183 (62.5%)	105 (62.9%)	78 (61.9%)	0.020
Broad microbiological category among the full cohort				
S. aureus	19 (6.5%)	12 (7.2%)	7 (5.6%)	0.067
Coagulase-negative/other staphylococci	63 (21.5%)	37 (22.2%)	26 (20.6%)	0.037
Streptococcus spp.	25 (8.5%)	17 (10.2%)	8 (6.3%)	0.139
Enterococcus spp.	20 (6.8%)	10 (6.0%)	10 (7.9%)	0.077
Gram-negative organisms	17 (5.8%)	10 (6.0%)	7 (5.6%)	0.019
Fungal organisms	2 (0.7%)	1 (0.6%)	1 (0.8%)	0.023
Other organisms	4 (1.4%)	1 (0.6%)	3 (2.4%)	0.148
Polymicrobial/mixed cultures	26 (8.9%)	17 (10.2%)	9 (7.1%)	0.108
Culture positive, organism not specified	7 (2.4%)	0 (0.0%)	7 (5.6%)	0.343

Footnote: Data are presented as n (%), with percentages calculated using the full cohort or calendar-period cohort as the denominator. Blood-culture-positive cases were assigned to mutually exclusive broad microbiological categories. Cultures containing organisms from more than one broad microbiological group were classified as polymicrobial/mixed; multiple organisms belonging to the same broad group were retained within that group. The S. aureus category includes MSSA, MRSA, and explicitly documented S. aureus in cultures without organisms from another broad microbiological group. Coagulase-negative/other staphylococci included documented KNS/CoNS, MRKNS, MSKNS, MRSE, MSSE, S. epidermidis, S. hominis, and related staphylococcal isolates. Gram-negative organisms included Escherichia coli, Klebsiella, Pseudomonas, Enterobacter, Acinetobacter, Achromobacter, Stenotrophomonas, Brucella, and other documented Gram-negative isolates. Seven culture-positive cases, all in the 2018–2026 period, did not have an organism specified in the dataset. Standardized mean differences are presented as absolute values and are descriptive rather than inferential.

Supplementary Table S3. Infective endocarditis phenotype and structural characteristics according to calendar period

Characteristic	Overall (N=293)	2008–2017 (N=167)	2018–2026 (N=126)	Absolute SMD
**Infective endocarditis phenotype**				
Native-valve IE	175 (59.7%)	107 (64.1%)	68 (54.0%)	0.207
Prosthetic-valve IE	37 (12.6%)	16 (9.6%)	21 (16.7%)	0.211
CIED-related IE	68 (23.2%)	37 (22.2%)	31 (24.6%)	0.058
Mixed/combined IE	10 (3.4%)	5 (3.0%)	5 (4.0%)	0.053
Unknown IE phenotype	3 (1.0%)	2 (1.2%)	1 (0.8%)	0.041
**Vegetation location**				
Mitral	88 (30.0%)	47 (28.1%)	41 (32.5%)	0.096
Aortic	65 (22.2%)	45 (26.9%)	20 (15.9%)	0.272
Tricuspid	20 (6.8%)	11 (6.6%)	9 (7.1%)	0.022
Device/lead	66 (22.5%)	35 (21.0%)	31 (24.6%)	0.087
Multisite/combined	46 (15.7%)	22 (13.2%)	24 (19.0%)	0.160
Pulmonary	1 (0.3%)	1 (0.6%)	0 (0.0%)	0.110
No vegetation	3 (1.0%)	2 (1.2%)	1 (0.8%)	0.041
Unknown/missing location	4 (1.4%)	4 (2.4%)	0 (0.0%)	0.222
Maximum vegetation size, mm*	15 [11–20]	15 [11–20]	15 [11–21]	0.103
**Structural and clinical characteristics**				
Severe regurgitation	129 (44.0%)	70 (41.9%)	59 (46.8%)	0.099
Abscess	14 (4.8%)	10 (6.0%)	4 (3.2%)	0.135
Perforation	28 (9.6%)	14 (8.4%)	14 (11.1%)	0.092
Dehiscence	13 (4.4%)	6 (3.6%)	7 (5.6%)	0.094
Septic shock	72 (24.6%)	41 (24.6%)	31 (24.6%)	0.001
Previous infective endocarditis	6 (2.0%)	1 (0.6%)	5 (4.0%)	0.227
Oncological comorbidity	14 (4.8%)	4 (2.4%)	10 (7.9%)	0.252

Footnote: Data are presented as n (%) or median [interquartile range]. Standardized mean differences (SMDs) are presented as absolute values and are descriptive rather than inferential. For categorical variables, SMDs represent each category versus all other categories. Maximum vegetation size was available in 282 patients and was harmonized to millimeters using the largest recorded dimension. CIED denotes cardiac implantable electronic device. Structural complications such as abscess, perforation, and dehiscence were recorded during the index IE episode; because precise timing relative to the prediction time was not consistently available, these variables were not incorporated into Model A.

Supplementary Table S4. Surgical indications and treatment pathways

Panel A. Surgical treatment pathway

Treatment pathway	N (%)
Surgery within 180 days	122 (41.6%)
Surgery after 180 days	8 (2.7%)
No surgery during follow-up despite a record of surgery being recommended but not performed	62 (21.2%)
No surgery during follow-up and no record of recommended surgery not being performed	101 (34.5%)
Total	293 (100%)

Footnote: Treatment pathways are mutually exclusive. Overall, 130 patients underwent surgery during follow-up, of whom 122 underwent surgery within 180 days and 8 underwent surgery after 180 days. One additional patient had an initial record of recommended surgery not being performed but subsequently underwent surgery after 180 days; this patient is classified in the "surgery after 180 days" category in Panel A. CIED system extraction recorded as a surgical procedure was included in the cardiac-surgery exposure, whereas extraction without a recorded cardiac surgical procedure was not classified as cardiac surgery.

Panel B. Reasons why recommended surgery was not performed

Among the 63 patients with a record indicating that surgery had been recommended but was not performed at the relevant clinical decision point:

Recorded reason	N (%)
Death before surgery	34 (54.0%)
Patient declined surgery	21 (33.3%)
Clinical regression with antimicrobial therapy	4 (6.3%)
Medical management because of overall clinical condition	1 (1.6%)
Surgery deferred until after pregnancy	1 (1.6%)
Reason unspecified/coded as 0	2 (3.2%)
Total	63 (100%)

Footnote: Reasons are reported as recorded in the source dataset. The variable describing why surgery was not performed contains post-baseline clinical information, including death before surgery, and was therefore used only for descriptive characterization and not as a predictor or adjustment variable.

Panel C. Recorded surgical indications

At least one surgical indication was recorded in 205 of 293 patients (70.0%). Multiple indications could coexist.

Recorded surgical indication	N (% of patients with ≥1 recorded indication)
Vegetation size	156 (76.1%)
Severe regurgitation	114 (55.6%)
Rupture	6 (2.9%)
Heart failure	4 (2.0%)
Fungal infection	3 (1.5%)
Abscess	1 (0.5%)

Footnote: Surgical indications were coded according to the source dataset and were not mutually exclusive; therefore percentages do not sum to 100%. Four patients had missing surgical-indication data, and 84 had no coded surgical indication. Because the dataset did not consistently provide the exact time at which each surgical indication became apparent relative to the index date, these variables were not treated as baseline predictors or confounders in the primary models.

Supplementary Table S5. Landmark analyses of surgery and subsequent 180-day mortality

Landmark	N at risk	Subsequent deaths	Operated by landmark	Crude HR (95% CI)	p-value	Adjusted HR (95% CI)	p-value
Day 14	259	71	34	0.25 (0.08–0.81)	0.020	0.37 (0.11–1.19)	0.096
Day 30	243	56	63	0.37 (0.17–0.82)	0.014	0.51 (0.23–1.17)	0.113

Footnote: Landmark analyses condition on survival to the specified landmark and therefore address a different estimand from the time-dependent Cox analysis. Early deaths before the landmark are necessarily excluded.

Supplementary Table S6. Proportional-hazards diagnostics for the primary Cox models

Variable	Model A p-value	Model B p-value
Age	0.582	0.590
NYHA functional class	0.034	0.039
Hemoglobin	0.413	0.401
BUN	0.755	0.750
LVEF	0.171	0.173
Severe regurgitation	0.612	0.660
Log-transformed CRP	0.033	0.033
Surgery within 180 days	—	<0.001
GLOBAL	0.045	0.002

Footnote: Proportional-hazards assumptions were assessed using scaled Schoenfeld residuals (cox.zph, Kaplan–Meier time transformation). The fixed future-surgery variable in Model B showed marked non-proportionality (exact p=0.00053); the global Model B test was p=0.00162.

Supplementary Table S7. Fixed-horizon 180-day logistic-regression sensitivity analysis

Metric	Model A	Model B
Patients with ascertainable 180-day status	261	261
180-day deaths	88	88
AUC	0.812	0.812
Brier score	0.16174	0.16171

Additional model comparison:

Comparison	Estimate
Surgery OR (95% CI)	0.86 (0.44–1.67)
Surgery coefficient P value	0.655
Nested likelihood-ratio test P value	0.655
DeLong test for AUC difference P value	0.891
AUC difference, B−A	+0.00039
95% CI for AUC difference	−0.00601 to +0.00523

Footnote: This fixed-horizon sensitivity analysis was performed among patients with ascertainable 180-day status and does not rely on the proportional-hazards assumption. Patients censored before 180 days without an event were excluded from this analysis.

Supplementary Table S8. Horizon-specific predictive performance of Model A and Model B

Panel A. Apparent performance

Horizon	Model A AUC	Model B AUC	ΔAUC, percentage points (95% CI)	p-value	Model A Brier	Model B Brier
30 days	0.856	0.863	+0.72 (−1.71 to 3.15)	0.563	0.0710	0.0685
90 days	0.815	0.818	+0.29 (−2.34 to 2.91)	0.831	0.1286	0.1270
180 days	0.792	0.793	+0.06 (−1.75 to 1.87)	0.949	0.1633	0.1636

Panel B. Bootstrap cross-validation

Horizon	Model A CV-AUC	Model B CV-AUC	ΔAUC, percentage points (95% CI)	Model A CV-Brier	Model B CV-Brier
30 days*	0.800	0.797	−0.33 (−10.03 to 4.09)	0.0867	0.0860
90 days	0.787	0.782	−0.43 (−5.51 to 2.43)	0.1451	0.1454
180 days	0.759	0.749	−0.95 (−5.68 to 1.40)	0.1798	0.1824

Footnote: *Model B incorporated surgery occurring within the corresponding prediction horizon.* Thirty-day bootstrap estimates should be interpreted cautiously because only two 30-day deaths occurred among patients undergoing surgery within 30 days, resulting in repeated convergence/separation warnings during resampling.

Supplementary Table S9. Temporal case-mix and calibration diagnostics

Panel A. Case-mix comparison in the complete-case temporal cohorts

Characteristic	2008–2017 derivation (N=155)	2018–2026 validation (N=123)	Absolute SMD
Age, years, mean	54.16	57.41	0.196
NYHA functional class, mean	1.890	1.683	0.192
BUN, mg/dL, mean	28.29	31.16	0.112
Log-transformed CRP, mean	4.530	4.249	0.270
Severe regurgitation	41.9%	47.2%	0.105
Surgery within 180 days	43.2%	40.7%	0.052
Prognostic index from derivation Model A, mean	0.0765	−0.0587	0.137

Panel B. Temporal transportability and calibration diagnostics

Metric	Result
Derivation cohort, N	155
Derivation cohort 180-day deaths	47
Derivation cohort censored alive before 180 days	8
Derivation cohort 180-day KM mortality	31.1% (95% CI 23.2–38.1)
Validation cohort, N	123
Validation cohort 180-day deaths	41
Validation cohort censored alive before 180 days	9
Validation cohort 180-day KM mortality	34.7% (95% CI 25.5–42.8)
Validation cohort mean predicted 180-day mortality	27.3%
Absolute observed–predicted difference	+7.5 percentage points (bootstrap 95% CI −2.4 to +18.8)
Observed-to-expected risk ratio	1.27 (bootstrap 95% CI 0.93–1.88)
Temporal validation AUC, Model A	0.731 (95% CI 0.622–0.839)
Temporal calibration slope	1.025 (95% CI 0.711–1.338)
Later-era HR with derivation prognostic index used as offset	1.47 (95% CI 0.97–2.24)
P value for later-era offset effect	0.070

Footnote: Temporal validation used models fitted exclusively in the 2008–2017 complete-case derivation cohort and applied without refitting to the 2018–2026 complete-case validation cohort. The prognostic index did not indicate a more adverse measured case-mix in the later cohort despite higher observed mortality. The later-era offset estimate is compatible with temporal baseline-risk drift but does not establish it statistically; unmeasured case-mix, referral patterns, microbiology, complications, treatment changes, and outcome ascertainment may also contribute. Observed mortality was estimated using the Kaplan–Meier method. The O/E risk ratio was calculated as Kaplan–Meier observed 180-day mortality divided by mean predicted 180-day mortality. Confidence intervals for the absolute observed–predicted difference and O/E risk ratio were obtained from 2,000 independent bootstrap resamples of the derivation and validation cohorts, with Model A refitted in each derivation resample and applied without recalibration to the corresponding validation resample. The 17 patients censored alive before 180 days (8 in the derivation cohort and 9 in the validation cohort) were foreign nationals for whom reliable national vital-status registry linkage was unavailable.

Supplementary Table S10. Sensitivity of time-dependent and phase-specific surgery estimates to same-day surgery/death handling

Panel A. Overall time-dependent surgery analysis

Same-day convention	Crude HR (95% CI)	Crude p-value	Adjusted HR (95% CI)	Adjusted p-value
Same-day deaths classified as pre-surgery	0.85 (0.51–1.40)	0.524	1.29 (0.75–2.20)	0.357
Same-day deaths classified as postoperative	1.16 (0.72–1.87)	0.550	1.84 (1.10–3.09)	0.021
Same-day surgery/death records excluded	0.93 (0.56–1.55)	0.787	1.44 (0.83–2.49)	0.196

Panel B. Phase-specific mortality according to alternative same-day conventions

Same-day convention	Preoperative deaths / person-days	Postop 0–14 deaths / person-days	Postop >14 deaths / person-days	Adjusted HR, postop 0–14	Adjusted HR, postop >14
Same-day death = preoperative	65 / 23,119.5	13 / 1,476.0	10 / 11,922.0	3.19 (1.70–5.97)	0.64 (0.31–1.32)
Same-day death = postoperative	60 / 23,117.0	18 / 1,478.5	10 / 11,922.0	5.00 (2.84–8.81)	0.71 (0.34–1.48)
Same-day records excluded	60 / 22,900.5	13 / 1,476.0	10 / 11,922.0	3.54 (1.88–6.69)	0.70 (0.34–1.47)

Footnote: Five patients had surgery and death recorded on the same calendar day. Because within-day ordering was unavailable, three alternative specifications were examined: same-day death treated as occurring before surgery, same-day death treated as postoperative, and exclusion of the same-day records. Person-time is reported in person-days. Phase-specific hazard ratios compare each postoperative state with the preoperative state and are adjusted for age, NYHA functional class, hemoglobin, BUN, LVEF, severe regurgitation, and log-transformed CRP. These analyses are exploratory and describe associations with postoperative state; they should not be interpreted as causal effects of surgery.

Supplementary Table S11. Sensitivity analysis excluding patients with CIED-related infective endocarditis

Analysis	N	180-day deaths	Surgery ≤180 d	Adjusted HR (95% CI)	p value
Fixed baseline surgery	211	70	80	0.50 (0.27–0.93)	0.028
Time-dependent surgery	211	70	80	1.43 (0.77–2.66)	0.263

Footnote: Patients with CIED-related IE were excluded. To maintain comparability with the primary fixed-versus-time-dependent analysis, four non-CIED patients with surgery and death recorded on the same calendar day were also excluded, leaving 211 patients. Both models used the same adjustment set: age, NYHA functional class, hemoglobin, BUN, LVEF, severe regurgitation, and log-transformed CRP.

Supplementary Table S12. Multiple-imputation sensitivity analysis for missing baseline predictors

Analysis	N	Surgery HR (95% CI)	p-value
Complete-case adjusted fixed-surgery model	278	0.71 (0.44–1.17)	0.181
Multiple-imputation adjusted fixed-surgery model	293	0.67 (0.41–1.11)	0.116

Multiple-imputation model comparison	Result
Number of imputations	50
D1 statistic	2.52
df	1
D1 P value	0.116
Relative increase in variance	0.00047

Footnote: Fifteen patients (5.1%) had missing data in at least one primary model covariate: CRP in 13 patients, BUN in 1, and LVEF in 1. Missing continuous predictors were imputed using predictive mean matching. The imputation model included baseline predictors, the event indicator, and the Nelson–Aalen cumulative hazard. Subsequent surgery was deliberately excluded as an imputation predictor to avoid introducing post-baseline treatment information into the baseline predictor set used for Model A. Estimates were combined using Rubin’s rules.
